# Bortezomib/docetaxel combination therapy in patients with anthracycline-pretreated advanced/metastatic breast cancer: a phase I/II dose-escalation study

**DOI:** 10.1038/sj.bjc.6604347

**Published:** 2008-04-29

**Authors:** A Awada, J Albanell, P A Canney, L Y Dirix, T Gil, F Cardoso, P Gascon, M J Piccart, J Baselga

**Affiliations:** 1 Medical Oncology Clinic, Institut Jules Bordet, Brussels, Belgium; 2 Hospital Clinic, Servicio de Oncologia Médica, IDIBAPS, Barcelona, Spain; 3 West of Scotland Clinical Trials Unit, Beatson Oncology Centre, Glasgow, UK; 4 Oncology Centre, AZ Sint Augustinus, Antwerp, Belgium; 5 Hospital Universitario Vall d'Hebron, Barcelona, Spain

**Keywords:** anthracycline-pretreated, breast cancer, bortezomib, docetaxel, metastatic, proteasome inhibitor

## Abstract

The aim of this study was to determine the dose-limiting toxicities (DLTs) and maximum tolerated dose (MTD) of bortezomib plus docetaxel in patients with anthracycline-pretreated advanced/metastatic breast cancer. Forty-eight patients received up to eight 21-day cycles of docetaxel (60–100 mg m^−2^ on day 1) plus bortezomib (1.0–1.5 mg m^−2^ on days 1, 4, 8, and 11). Pharmacodynamic and pharmacokinetic analyses were performed in a subset of patients. Five patients experienced DLTs: grade 3 bone pain (*n*=1) and febrile neutropenia (*n*=4). The MTD was bortezomib 1.5 mg m^−2^ plus docetaxel 75 mg m^−2^. All 48 patients were assessable for safety and efficacy. The most common adverse events were diarrhoea, nausea, alopecia, asthenia, and vomiting. The most common grade 3/4 toxicities were neutropenia (44%), and febrile neutropenia and diarrhoea (each 19%). Overall patient response rate was 29%. Median time to progression was 5.4 months. In patients with confirmed response, median time to response was 1.3 months and median duration of response was 3.2 months. At the MTD, response rate was 38%. Pharmacokinetic characteristics of bortezomib/docetaxel were comparable with single-agent data. Addition of docetaxel appeared not to affect bortezomib inhibition of 20S proteasome activity. Mean alpha-1 acid glycoprotein concentrations increased from baseline at nearly all time points across different bortezomib dose levels. Bortezomib plus docetaxel is an active combination for anthracycline-pretreated advanced/metastatic breast cancer. The safety profile is manageable and consistent with the side effects of the individual agents.

Anthracyclines and taxanes are widely used in treating patients with metastatic breast cancer. In anthracycline-pretreated advanced/metastatic breast cancer, docetaxel administered once every 3 weeks is more active than the same schedule of paclitaxel (Jones *et al*, 2005). The efficacy of docetaxel as a single agent (Nabholtz *et al*, 1999; Sjostrom *et al*, 1999; Jones *et al*, 2005) or in combination with cytotoxic agents (O'Shaughnessy *et al*, 2002; Chan *et al*, 2005) has been established in phase III trials in this setting. The standard dose of single-agent docetaxel is 75–100 mg m^−2^ every 21 days. In combination, the dose is reduced, typically to 75 mg m^−2^ (Aventis Pharmaceuticals Inc, 2005). Dose reduction potentially reduces efficacy; therefore, careful identification of dosing regimens that provide acceptable safety profiles without compromising efficacy is essential.

Combining agents with unique modes of action may improve outcomes and overcome chemoresistance without significantly increasing toxicity. Docetaxel acts by disrupting the microtubular network essential for cellular functions (Bissery *et al*, 1995; Eisenhauer and Vermorken, 1998). Bortezomib, which is approved for treating multiple myeloma and mantle cell lymphoma patients who have received at least one prior therapy (Kane *et al*, 2006), acts by inhibiting the 26S proteasome, the degradative enzyme complex involved in the catabolism of numerous intracellular regulatory proteins, including NF-*κ*B (nuclear factor-*κ*B)-inhibitor I*κ*B*α*, p53, p21, and p27 (Adams, 2002; Cusack, 2003; Lenz, 2003; Boccadoro *et al*, 2005). Malignancies with high concentrations of activated NF-*κ*B, such as breast cancer, are logical targets for agents that interrupt this pathway (Orlowski and Dees, 2003). Mutations in the tumour suppressor gene p53 occur in 20–40% of sporadic breast cancers (Osin and Lakhani, 1999) and are associated with a poor prognosis (Pharoah *et al*, 1999; Overgaard *et al*, 2000) and poor response to treatment with certain chemotherapeutic and hormonal agents (Berns *et al*, 2000; Kandioler-Eckersberger *et al*, 2000). The cyclin-dependent kinase inhibitors p21 and p27 also play important roles in breast cancer (Osin and Lakhani, 1999), supporting the investigation of bortezomib in breast malignancies.

Bortezomib has demonstrated cytotoxic activity in breast, lung, pancreatic, prostate, and head and neck tumour models *in vivo* (Adams *et al*, 1999; Teicher *et al*, 1999; Shah *et al*, 2001; Sunwoo *et al*, 2001; Nawrocki *et al*, 2002; Williams *et al*, 2003; Ikezoe *et al*, 2004). Preliminary *in vitro* and *in vivo* studies in a range of solid tumours demonstrated an additive antitumour effect of bortezomib with standard cytotoxic agents, including docetaxel (Gumerlock *et al*, 2002, 2004; Mack *et al*, 2003; Nawrocki *et al*, 2004; Farneth *et al*, 2005). Bortezomib 1 mg kg^−1^ combined with docetaxel 5 mg kg^−1^ was active in pancreatic xenograft models (Nawrocki *et al*, 2004). Interestingly, sequential administration led to greater tumour growth inhibition than with either agent alone (Gumerlock *et al*, 2004). Preclinical studies in prostate and lung cancer models indicated that the sequence docetaxel → bortezomib was more effective than bortezomib → docetaxel (Gumerlock *et al*, 2002; Farneth *et al*, 2005). A possible explanation is that bortezomib promotes cell cycle arrest before the M phase, consequently interfering with docetaxel-induced apoptosis (Nawrocki *et al*, 2004). Additionally, *in vitro* studies have shown that docetaxel is extensively plasma protein-bound, especially to alpha-1 acid glycoprotein (AAG), albumin, and lipoproteins (Aventis Pharmaceuticals Inc, 2005). As AAG is an acute-phase protein that may be elevated by IL (interleukin)-6, which is inhibited by bortezomib (Hideshima *et al*, 2001), bortezomib may decrease AAG concentrations and improve docetaxel efficacy.

In clinical studies in patients with solid tumours, bortezomib/docetaxel combination therapy demonstrated encouraging activity, and the side effects were predictable and manageable (Meluch *et al*, 2005; Fanucchi *et al*, 2006; Lara *et al*, 2006; Dreicer *et al*, 2007). There is no evidence of a drug interaction between bortezomib and docetaxel (Messersmith *et al*, 2006).

Based on the known single-agent antitumour activity of bortezomib and docetaxel, and their additive efficacy in preclinical models, complementary mechanisms of action (Nawrocki *et al*, 2004), different toxicity profiles, and feasibility in clinical studies in various solid tumours, we conducted the current study to explore the potential of bortezomib/docetaxel combination therapy in patients with advanced/metastatic breast cancer. Our primary aim was to identify the most appropriate regimen for further evaluation.

## PATIENTS AND METHODS

### Study objectives

The primary objective was to establish the dose-limiting toxicity (DLT) and maximum tolerated dose (MTD) of bortezomib/docetaxel combination therapy in patients with advanced and/or metastatic breast cancer that had previously been treated with anthracyclines. Secondary objectives were to assess tumour response rate (complete response (CR), partial response (PR), or stable disease (SD)), time to response, duration of response, and time to disease progression (TTP); evaluate disposition profiles of docetaxel and bortezomib administered in combination using pharmacokinetic analyses; evaluate pharmacodynamic properties of bortezomib in combination with docetaxel using 20S proteasome inhibition assay (Lightcap *et al*, 2000); and investigate the possible influence of bortezomib/docetaxel combination treatment on serum concentrations of AAG.

### Eligibility

All study participants provided written, informed consent. Patients who had advanced/metastatic breast cancer, had received at least one anthracycline-containing regimen, were aged ⩾18 years, and had a Karnofsky Performance Status ⩾80% (ECOG 0–1) were considered eligible. Prior treatment with endocrine or biologic agents was permitted. Patients were excluded if they had previously received docetaxel or paclitaxel as adjuvant treatment within the previous year or for metastatic disease at any time; radiotherapy to >35% of bone marrow (e.g., pelvic radiation), major surgery, or chemotherapy within 4 weeks of enrollment; nitrosoureas within 6 weeks or antibody therapy within 8 weeks of enrollment; or high-dose chemotherapy and peripheral blood stem cell transplantation at any time. Patients were excluded if they had grade ⩾1 peripheral neuropathy; abnormal laboratory values within 2 weeks of enrollment; history of severe hypersensitivity reaction to docetaxel; not recovered from all toxic effects (except alopecia) of previous chemotherapy, radiotherapy, or antibody therapy. The following were prohibited during the study treatment: any investigational agent other than bortezomib, haematopoietic growth factors during the first cycle (except for haematologic DLT, or continued erythropoietin for pre-existing anaemia), immunotherapeutic agents, steroids (except dexamethasone), chemotherapeutic agents other than docetaxel, radiotherapy, or surgery for cancer.

### Study design

This prospective, phase I/II, open-label dose-escalation study was conducted at six centers in Europe (Belgium, Spain, UK) in accordance with the International Conference on Harmonisation for Good Clinical Practice. The protocol and informed consent were approved by the Institutional Review Board at each clinical site before study initiation.

Each 3-week treatment cycle consisted of docetaxel (Taxotere®; Sanofi-Aventis, Paris, France) infusion on day 1 and bortezomib (Velcade®; Millennium Pharmaceuticals Inc., Cambridge, MA, USA and Johnson & Johnson Pharmaceuticals, Rariman, NY, USA, Research and Development, L.L.C) injections on days 1, 4, 8, and 11. Bortezomib was administered 1 h after docetaxel. Treatment comprised eight cycles. Docetaxel was administered at doses of 60, 75, or 100 mg m^−2^ with standard oral dexamethasone premedication (six 8-mg doses administered the night before, morning of, immediately before, evening after, and morning and evening of day 2 after each docetaxel infusion). Bortezomib doses were 1.0, 1.3, or 1.5 mg m^−2^, based on tolerability and efficacy in heavily pretreated patients with solid tumours. All patients received appropriate supportive therapy. Patients achieving a tumour response at the end of the treatment could receive further treatment at the investigator's discretion. Patients with progressive disease discontinued treatment.

Patients were evaluated for toxicities before each scheduled study drug dose. Adverse events (AEs) were graded according to the National Cancer Institute Common Toxicity Criteria (version 2.0). The following AEs during the first cycle were considered DLTs: platelet count ⩽25 × 10^9^ l^−1^; febrile neutropenia (absolute neutrophil count [ANC]<1 × 10^9^ l^−1^ with temperature ⩾38.5°C); ANC<0.5 × 10^9^ l^−1^ for days 1–7, or <0.2 × 10^9^ l^−1^ without fever for ⩾7 days starting on or after day 8; any other grade 4 haematologic toxicity; grade 2 peripheral neuropathy; any grade 3 or 4 non-haematologic toxicity (except inadequately treated nausea, vomiting, and diarrhoea). In patients experiencing grade 4 haematologic or grade 3/4 non-haematologic toxicity, the start of the next cycle was delayed for up to 2 weeks or bortezomib therapy was interrupted for up to 2 weeks until toxicity returned to baseline or better. Treatment was re-initiated at a reduced dose (25% dose reduction of the drug considered by the investigator to have caused the toxicity). A maximum of two dose reductions for each drug was recommended. If toxicity did not resolve, the patient was excluded from further study.

Doses of both agents were reduced if patients experienced grade 2 neuropathic pain or peripheral neuropathy. In patients with either toxicity at grade 3 intensity or both toxicities at grade 2, bortezomib treatment was interrupted until resolution to grade ⩽1, when dose was reduced, and at resolution, treatment was continued using weekly bortezomib (days 1 and 8 only). In patients experiencing grade 4 neuropathic pain or peripheral neuropathy, or both toxicities with grade 3 intensity, bortezomib and docetaxel were discontinued.

Doses were escalated in a stepwise fashion if fewer two out of three patients in a dose cohort experienced a DLT. The MTD was defined as the dose below that causing DLTs in at least two out of three patients. Up to 10 additional patients were to be enrolled at the MTD.

### Study assessments

Karnofsky Performance Status assessments, physical examinations, and laboratory sampling were undertaken during screening, during the first cycle, at the end-of-therapy visit (10 days after the last dose of study drug), and at the end-of-study visit (3 weeks after the end-of-therapy visit).

Lesions were assessed by CT/MRI every 6 weeks using the Response Evaluation Criteria in Solid Tumours (Therasse *et al*, 2000). Response was confirmed 6 weeks later in patients with CR/PR. Efficacy was also assessed based on the expression of tumour marker CA15.3, Karnofsky Performance Status, and C-reactive protein, IL-6, and AAG concentrations. Blood samples were collected at screening, on day 1 of cycles 3, 5, and 7 (and all odd-numbered cycles thereafter in responding patients receiving further treatment), and at the end-of-study visit.

In patients participating in pharmacokinetic analysis, blood samples were taken on day 1, cycle 1, to determine the plasma concentration time profile of docetaxel and bortezomib. Samples were collected immediately before docetaxel infusion and bortezomib injection and at prespecified intervals on day 1. Samples were also taken for bortezomib pharmacokinetic analysis on day 11 of cycle 1. Whole-blood samples were taken for pharmacodynamic analysis using 20S proteasome inhibition assay (Lightcap *et al*, 2000) immediately before and 1 h after bortezomib dosing on days 1 and 11 of cycles 1 and 2, and on days 2 and 12 of cycle 1, in a subset of patients participating in pharmacokinetic analysis. Samples for determination of AAG concentrations were collected at screening and before docetaxel administration on day 1 of cycles 1, 2, 4, and 6.

### Statistical analysis

Statistical analyses were primarily descriptive; the aim of the study was to establish the MTD of bortezomib/docetaxel combination therapy. Planned enrollment was up to 70 patients.

Safety and efficacy were evaluated in all patients who received any amount of either study drug. Patients who underwent dose reduction were analysed in the dose group in which they were initially treated. The MTD-evaluable population included all patients in the dose-escalation phase with sufficient safety assessments during cycle 1 to determine whether a DLT occurred. Patients were excluded from the MTD-evaluable population if they had discontinued during cycle 1 for reasons other than DLTs or had received alternate antineoplastic therapies during that period.

## RESULTS

### Patient characteristics and disposition

Forty-eight patients were enrolled (47 females and 1 male); all received at least one dose of docetaxel or bortezomib. Patient characteristics are summarised in Table 1 (sites of metastases at study entry: bone (*n*=23), liver (*n*=12), lung (*n*=4), skin and soft tissues (*n*=8), nodes (*n*=6), and others (*n*=8)). The median duration of treatment was 95 days (range: 11–179) or 4 cycles (range: 1–9); 12 patients (25%) completed all 8 cycles of planned therapy.

### Identification of MTD

Table 2 summarises patients treated and DLTs observed at each dose level. No DLTs occurred at the first two dose levels (1.0/60 and 1.0/75 mg m^−2^). At the 1.3/75 mg m^−2^ dose level, one patient developed grade 3 bone pain, which is considered a DLT and possibly related to an induced flare of pain associated with bone metastases. One patient each at the 1.0/100 and 1.5/75 mg m^−2^ dose levels, and two at the 1.3/100 mg m^−2^ dose level developed febrile neutropenia. Consequently, the MTD was defined as bortezomib 1.5 mg m^−2^ plus docetaxel 75 mg m^−2^.

### Safety

All 48 patients experienced at least 1 AE, most commonly diarrhoea, nausea, alopecia, asthenia, and vomiting (Table 3). Grade 3/4 AEs occurred in 37 patients (77%) and were considered drug-related in 34 (71%) patients. The most common grade 3/4 AEs were neutropenia, febrile neutropenia, and diarrhoea (Table 3).

Eighteen (50%) out of the 36 patients who withdrew prematurely had unacceptable AEs listed as the primary reason for treatment discontinuation: 4 at the bortezomib 1.0 mg m^−2^ dose level, 6 at the 1.3 mg m^−2^ dose level, and 8 at the 1.5 mg m^−2^ dose level. Events leading to discontinuation included peripheral neuropathy (nine patients), neuralgia (four patients), paraesthesia and diarrhoea (two patients each), and neutropenia (one patient). Twelve patients (33%) withdrew because of progressive disease. Dose reductions were most common in the second and third cycles of treatment (19 and 15% patients, respectively). Two patients died on study, one due to cardiac tamponade 3 days after the last dose of study drug and the other from disease progression 22 days after the last dose of study drug.

### Efficacy

Overall, 29% (14 out of 48) patients achieved a confirmed PR and 56% (27 out of 48) achieved SD. There were no CRs. At MTD (1.5/75 mg m^−2^), 38% (6 out of 16) patients achieved a PR, with an overall clinical benefit (PR+SD) rate of 75% (12 out of 16 patients; Table 4).

Among 14 patients achieving confirmed PR, median time to response was 39 days (1.3 months) and median duration of response was 96.5 days (3.2 months). Among 27 patients achieving SD, median duration of disease stabilisation was 100.0 days (3.3 months). Median TTP for all patients was 164 days (5.4 months). Data are shown by bortezomib dose level in Table 4.

### Pharmacokinetics

Bortezomib plasma profiles exhibited an initial rapid distribution phase followed by a slower elimination phase, demonstrating apparent multi-exponential decay characteristics, similar to those reported previously. Bortezomib was eliminated from plasma with similar terminal half-lives across dose levels, with mean values of 15–18 h following the first dose in patients with measurable plasma concentrations at 24 h post-dose (Table 5). Pharmacokinetic characteristics of bortezomib/docetaxel were comparable with single-agent data (Millennium Pharmaceuticals Inc.; Rosing *et al*, 2000).

### Pharmacodynamics

In total, 33 patients provided whole-blood samples for measurement of 20S proteasome inhibition at baseline. Inhibition of proteasome activity was greatest at 1 h post-dose (46.14–88.80%). Immediately prior to the dose on day 11, percent inhibition was 10.93–51.18%. Percent inhibition was greater in cycle 2 than cycle 1 for all dose groups. Coadministration of docetaxel with bortezomib did not appear to affect 20S proteasome inhibition by bortezomib. The degree of inhibition observed is similar to that observed in studies of bortezomib monotherapy (Aghajanian *et al*, 2002; Orlowski *et al*, 2002; Blaney *et al*, 2004; Papandreou *et al*, 2004; Hamilton *et al*, 2005).

**Figure 1:**
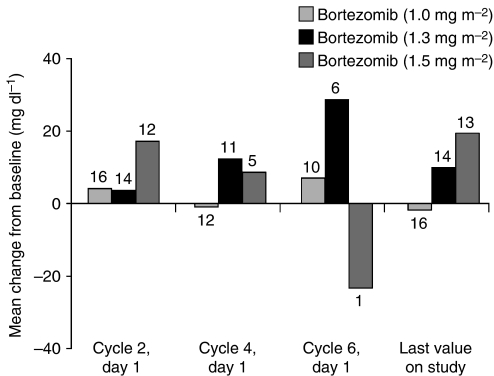
Mean change from baseline in alpha-1 acid glycoprotein concentrations by bortezomib dose level during the study. The number of patients available for each analysis are shown above or below each column; notably, the significant decrease from baseline seen at cycle 6, day 1, in the bortezomib 1.5 mg m^−2^ group arises from evaluation of data from only one patient.

Limited blood samples were collected for IL-6 and CRP analysis. Therefore, no analyses of these data were conducted. Mean AAG concentrations increased from baseline for nearly all time points across all three bortezomib dose levels (Figure 1).

## DISCUSSION

In this phase I/II study in patients with pretreated advanced/metastatic breast cancer, the MTD was bortezomib 1.5 mg m^−2^ on days 1, 4, 8, and 11, plus docetaxel 75 mg m^−2^ on day 1 of a 21-day cycle. The DLTs were bone pain and febrile neutropenia. The bortezomib dose was slightly higher than the approved 1.3 mg m^−2^ on the same schedule, but similar to that identified in phase I studies evaluating single-agent bortezomib administered on the same schedule (Aghajanian *et al*, 2002; Dy *et al*, 2005). Slightly higher doses were tolerated when bortezomib was administered weekly (Papandreou *et al*, 2004) or on days 1 and 4 every 14 days (Hamilton *et al*, 2005). In other combination dose-escalation studies in advanced non-small-cell lung cancer and other refractory solid tumours (Lara *et al*, 2006), MTDs were bortezomib 1.0 mg m^−2^ plus docetaxel 75 mg m^−2^ on the same schedule, and bortezomib 0.8 mg m^−2^ twice weekly for 2 weeks plus docetaxel 25 mg m^−2^ on days 1 and 8 of a 21-day cycle (Messersmith *et al*, 2006). The docetaxel dose in our study is consistent with that used in combination with chemotherapeutic agents in anthracycline-pretreated breast cancer (O'Shaughnessy *et al*, 2002; Chan *et al*, 2005).

The safety profile of the combination was consistent with that observed in phase II studies in other solid tumours evaluating bortezomib alone (Shah *et al*, 2004; Maki *et al*, 2005) or in combination with docetaxel (Meluch *et al*, 2005; Fanucchi *et al*, 2006; Dreicer *et al*, 2007). The most common grade 3/4 toxicities were neutropenia, febrile neutropenia, diarrhoea, and peripheral neuropathy. Dose modifications were implemented to try and prevent evolution of neurotoxicity and worsening of existing symptoms. Nevertheless, in these heavily pretreated patients, 15 patients discontinued treatment due to neurological complaints, a known side effect of bortezomib and, to a lesser extent, docetaxel. The high incidence of neutropenia was expected given the typically high incidence of neutropenia and febrile neutropenia with single-agent docetaxel (Nabholtz *et al*, 1999; O'Shaughnessy *et al*, 2002) and the partially overlapping side-effect profiles of the two agents. Supportive therapies and strategies for side-effect management can prevent worsening of these symptoms, thereby avoiding treatment delays or discontinuations (Colson *et al*, 2004). Notably, bortezomib-associated neutropenia has been shown to be transient and reversible (Lonial *et al*, 2005); consistent with this, only one patient discontinued the study drug due to neutropenia.

Bortezomib/docetaxel combination treatment demonstrated antitumour activity. The response rate was 29% and median TTP was 5.4 months. These data are similar to those reported for docetaxel (75 mg m^−2^) in combination with either gemcitabine or capecitabine, which have been compared in a phase III trial (Chan *et al*, 2005). Recent studies have shown response rates of 62.5% with docetaxel plus cisplatin (Lin *et al*, 2007) and 50% with docetaxel plus epirubicin (Hainsworth *et al*, 2006). The 38% response rate at the MTD compares favourably with docetaxel and paclitaxel administered as single agents in anthracycline-pretreated metastatic breast cancer: in a recent study comparing docetaxel and paclitaxel, response rates were 32 *vs* 25%, and median TTP was 5.7 *vs* 3.6 months, respectively (Jones *et al*, 2005). The activity demonstrated in the present study does not appear to arise from the mechanism proposed, by which bortezomib reduces AAG concentrations through inhibition of IL-6 and consequently increases docetaxel efficacy, as mean AAG concentrations increases from baseline at nearly all time points across bortezomib dose levels. Other cellular and molecular effects of bortezomib may be involved; recently published results demonstrating its differential effects in breast cancer cells may be relevant in designing mechanism-based combination treatments (Codony-Servat *et al*, 2006).

Bortezomib has been studied in combination with trastuzumab in HER2-positive breast cancer cell lines (Cardoso *et al*, 2006). Although its value in HER2-postive cells is well established, the response rate with single-agent trastuzumab in metastatic breast cancer is <40% (Vogel *et al*, 2002). A preclinical study in four cell lines demonstrated that bortezomib acts synergistically to increase the effect of trastuzumab in HER2-positive cells (Cardoso *et al*, 2006). The potential clinical application of this combination is currently under investigation in a phase I trial. Additionally, a phase II study is underway to investigate bortezomib plus pegylated liposomal doxorubicin in patients with anthracycline-pretreated metastatic breast cancer.

The baseline characteristics of patients in the present study were typical of patients with anthracycline-pretreated advanced/metastatic breast cancer. The p53 and estrogen receptor status were not recorded but may be of interest in future trials, as *in vitro* studies suggest proteasome inhibition is at least partially dependent on the p53 status in breast cancer (MacLaren *et al*, 2001), and estrogen-receptor-negative status plus a dysregulated I*κ*B*α*/NF-*κ*B system is associated with greater bortezomib antitumour activity (Tapia *et al*, 2005).

In conclusion, the study demonstrated that bortezomib and docetaxel combination therapy is feasible, tolerable, and active in anthracycline-pretreated metastatic breast cancer.

## Figures and Tables

**Table 1:** Patient demographics and baseline characteristics (*N*=48)

	**Bortezomib/docetaxel dose (mg m^−2^ dose^−1^)**								
	**1.0/60 (*n*=3)**	**1.0/75 (*n*=3)**	**1.0/100 (*n*=11)**	**1.0 subtotal (*n*=17)**	**1.3/75 (*n*=11)**	**1.3/100 (*n*=4)**	**1.3 subtotal (*n*=15)**	**1.5/75 (*n*=16)**	**Total (*N*=48)**
Mean age (years)	52	62	50	53	55	53	54	59	55
									
*Karnofsky performance score*									
80 (%)	0	1 (33)	3 (27)	4 (24)	4 (36)	1 (25)	5 (33)	6 (38)	15 (31)
90−100 (%)	3 (100)	2 (67)	8 (73)	13 (76)	7 (64)	3 (75)	10 (67)	10 (63)	33 (69)
Regional lymph node metastases at diagnosis^a^ (TNM stage N1, N2, N3)	3 (100)	1 (33)	5 (45)	9 (53)	6 (55)	3 (75)	9 (60)	13 (81)	31 (65)
Distant metastases at diagnosis^a^ (TNM stage M1)	1 (33)	2 (67)	1 (9)	4 (24)	0	0	0	3 (19)	7 (15)
Median number of prior chemotherapy regimens^b^, *n* (range)	2 (2–3)	3 (2–4)	3 (1–6)	3 (1–6)	3 (1–6)	3.5 (2–7)	3 (1–7)	4 (1–6)	3 (1–7)

TNM=tumour-node metastasis.

a Per protocol, all patients had metastatic disease at the time of enrollment for the study.

b Including neoadjuvant/adjuvant chemotherapy.

**Table 2:** Patients treated and DLTs observed by bortezomib and docetaxel dose level

**Bortezomib (mg m^−2^)**	**Docetaxel (mg m^−2^)**	**Treated *n***	**MTD evaluable^a^ *n***	**DLT**
1.0	60	3	3	—
1.0	75	3	3	—
1.0	100	11	7^b^	Febrile neutropenia (*n*=1)
1.3	75	11	10^c^	Grade 3 bone pain (*n*=1)
1.5	75	16	6^d^	Febrile neutropenia (*n*=1)
1.3	100	4	4	Febrile neutropenia (*n*=2)
Total		48	33	

MTD=maximum tolerated dose; DLT=dose-limiting toxicity.

a The MTD-evaluable population included all patients in the dose-escalation phase with sufficient safety assessments during cycle 1 to determine whether a DLT occurred.

b Three patients did not complete cycle 1 of treatment, and one patient was excluded due to pre-existing peripheral neuropathy.

c One patient had study drug held in cycle 1 and was not used in the determination of MTD.

d Ten additional patients enrolled following determination of dose level as MTD.

**Table 3:** Percentage of patients experiencing the most common AEs (all grades; ⩾25% of all patients) and grade 3 or 4 AEs (⩾5% of all patients)

	**Bortezomib/docetaxel doses (mg m^−2^ dose^−1^)**						
	**1.0/60 *n*=3**	**1.0/75 *n=*3**	**1.0/100 *n*=11**	**1.3/75 *n*=11**	**1.3/100 *n*=4**	**1.5/75 *n*=16**	**Total *n*=48**
*AE (all grades)*							
Diarrhoea	100	67	73	82	100	75	79
Nausea	100	67	64	73	50	56	65
Alopecia	33	67	55	64	75	50	56
Asthenia	33	67	55	82	50	44	56
Vomiting	67	33	36	82	25	50	52
Neutropenia	100	67	45	55	25	38	48
Myalgia	67	100	45	45	50	31	46
Anorexia	33	33	27	73	75	31	44
Peripheral neuropathy	0	67	36	64	50	31	42
Dysgeusia	33	67	45	64	50	13	40
Paresthesia	0	67	36	64	50	25	40
Fatigue	67	33	36	27	50	31	35
Arthralgia	67	67	27	45	25	19	33
Conjunctivitis	0	67	55	18	50	13	29
Headache	67	33	36	27	25	19	29
Constipation	33	0	27	45	25	19	27
Pyrexia	0	33	27	27	25	31	27
Mucosal inflammation	0	33	36	27	25	19	25
Neuralgia	0	33	36	36	25	13	25
							
*AE (grade 3/4)*							
Neutropenia	100	33	45	55	25	31	44
Febrile neutropenia	0	0	18	18	50	19	19
Diarrhoea	33	0	18	27	25	13	19
Peripheral neuropathy	0	0	9	9	0	19	10
Leukopenia	0	33	27	0	0	6	10
Asthenia	0	33	0	18	0	6	8
Fatigue	0	0	0	0	25	13	6
Neuralgia	0	0	0	0	25	13	6

AEs=adverse events.

**Table 4:** Summary of efficacy

	**Bortezomib/docetaxel dose (mg m^−2^ dose^−1^)**								
	**1.0/60 (*n*=3)**	**1.0/75 (*n*=3)**	**1.0/100 (*n*=11)**	**1.0 subtotal (*n*=17)**	**1.3/75 (*n*=11)**	**1.3/100 (*n*=4)**	**1.3 subtotal (*n*=15)**	**1.5/75 (*n*=16)**	**Total (*N*=48)**
*Best response* a									
CR/PR	0	2 (67)	1 (9)	3 (18)	3 (27)	2 (50)	5 (33)	6 (38)	14 (29)
SD	2 (67)	1 (33)	9 (82)	12 (71)	7 (64)	2 (50)	9 (60)	6 (38)	27 (56)
CR, PR, or SD	2 (67)	3 (100)	10 (91)	15 (88)	10 (91)	4 (100)	14 (93)	12 (75)	41 (85)
									
*Time to response (days)*									
*N*	0	2	1	3	3	2	5	6	14
Median (range)	NE	79.5 (77–82)	34.0 (34–34)	77.0 (34–82)	37.0 (34–77)	139.5 (90–189)	77.0 (34–189)	38.5 (31–80)	39.0 (31–189)
									
*Duration of response (days)*									
Median (range)	NE	95.5 (34–157)	130.0	130.0 (34–157)	121.0 (108–141)	29.5 (3–56)	108.0 (3–141)	84.5 (14–153)	96.5 (3–157)
									
*Duration of SD (days)*									
*N*	2	1	9	12	7	2	9	6	27
Median (range)	133.5 (77–190)	183.0	118.0 (35–190)	122.0 (35–190)	111.0 (41–195)	63.0 (53–73)	108.0 (41–195)	63.5 (28–80)	100.0 (28–195)
									
*Time to progression (days)*									
*N*/Censored^b^	3/2	3/1	11/5	17/8	11/6	4/2	15/8	16/8	48/24
Median	NE	182	183	182	175	146	175	98	164

CR=complete response; NE=not evaluable; PR=partial response; SD=stable disease.

a Confirmed or unconfirmed.

b Patients who had not progressed were censored on the end-of-study visit date or, if unavailable, date of last dose.

**Table 5:** Pharmacokinetic parameters of bortezomib and docetaxel by dose level

		**All values shown as mean (%CV)**							
	**Day**	***C***_**0**_ **(ng ml^−1^)**	**AUC**_**0–*t***_ **(h ng ml^−1^)**	**AUC**_**0–∞∞**_ **(h ng ml^−1^)**	***T***_**½,z**_ **(h)**	***T***_**½,z**_ **24-h (h)**	**CL (l h^−1^)**	***V***_**ss**_ **(l)**	***V***_**z**_ **(l)**
*Bortezomib dose (mg m* ^*−2*^ *)*									
1.0 (*n*=5)	1	208 (34)	43.0 (38)	54.6 (31)	9.15 (64)	15.1 (*n*=2)	33.7 (30)	222 (75)	408 (63)
1.3 (*n*=3)	1	137 (54)	35.5 (31)	49.0 (28)	14.2 (47)	18.1 (*n*=2)	51.0 (38)	596 (45)	924 (26)
1.5 (*n*=8)	1	268 (71)	69.0 (43)	85.3 (51)	6.9 (99)	17.8 (*n*=2)	36.9 (57)	209 (68)	282 (77)
1.0 (*n*=5)	11	552 (148)	112 (32)	133 (24)	12.2 (32)	14.3 (*n*=4)	13.5 (25)	151 (49)	237 (46)
1.3 (*n*=3)	11	237 (59)	90.5 (27)	111 (18)	12.2 (73)	17.1 (*n*=2)	21.0 (11)	237 (96)	374 (78)
1.5 (*n*=6)	11	153 (41)	103 (25)	161 (31)	23.6 (36)	23.6	16.0 (23)	391 (47)	538 (42)
									
*Docetaxel dose (mg m* ^*−2*^ *)*									
		***C***_**max**_ **(ng ml^−1^)**	**AUC**_**0–*t***_ **(h ng ml^−1^)**	**AUC**_**0–∞∞**_ **(h ng ml^−1^)**	***T***_**½,z**_ **(h)**	***T***_**½,z**_ **24-h (h)**	**CL (l h^−1^)**	***V***_**ss**_ **(l)**	***V***_**z**_ **(l)**
75 (*n*=7)	1	1620 (38)	1704 (50)	1847 (54)	10.4 (86)	17.6 (*n*=3)	85.2 (47)	180 (72)	920 (33)
100 (*n*=6)	1	2522 (51)	2547 (36)	2731 (34)	15.0 (42)	17.4 (*n*=5)	69.6 (31)	372 (85)	1514 (55)

*C*_0_=back-extrapolated time 0 plasma drug concentration (bortezomib alone); *C*_max_=observed maximum plasma drug concentration (docetaxel alone); AUC_0−*t*_=area under plasma concentration time curve from time 0 to last time point with measurable drug concentration; AUC_0−∞_=area under plasma concentration time curve extrapolated to infinity; *T*_½,z_=apparent plasma half-life; T_½,z_ (24-h)=apparent plasma half-life in patients with measurable plasma concentrations at 24 h post-dose; CL=total body clearance; CV=coefficient of variance; *V*_ss_=volume of distribution at steady state; *V*_z_=apparent volume of distribution in terminal elimination phase.

## References

[bib1] Adams J (2002) Development of the proteasome inhibitor PS-341. Oncologist 7: 9–16 10.1634/theoncologist.7-1-9 11854543

[bib2] Adams J, Palombella VJ, Sausville EA, Johnson J, Destree A, Lazarus DD, Maas J, Pien CS, Prakash S, Elliott PJ (1999) Proteasome inhibitors: a novel class of potent and effective antitumor agents. Cancer Res 59: 2615–2622 10363983

[bib3] Aghajanian C, Soignet S, Dizon DS, Pien CS, Adams J, Elliott PJ, Sabbatini P, Miller V, Hensley ML, Pezzulli S, Canales C, Daud A, Spriggs DR (2002) A phase I trial of the novel proteasome inhibitor PS341 in advanced solid tumor malignancies. Clin Cancer Res 8: 2505–2511 12171876

[bib4] Aventis Pharmaceuticals Inc. (2005) Taxotere (Prescribing Information). Bridgewater, NJ: Aventis Pharmaceuticals Inc.

[bib5] Berns EM, Foekens JA, Vossen R, Look MP, Devilee P, Henzen-Logmans SC, van Staveren IL, van Putten WL, Inganas M, Meijer-van Gelder ME, Cornelisse C, Claassen CJ, Portengen H, Bakker B, Klijn JG (2000) Complete sequencing of TP53 predicts poor response to systemic therapy of advanced breast cancer. Cancer Res 60: 2155–2162 10786679

[bib6] Bissery MC, Nohynek G, Sanderink GJ, Lavelle F (1995) Docetaxel (Taxotere): a review of preclinical and clinical experience. Part I: preclinical experience. Anticancer Drugs 6: 339–355, 363–8 7670132 10.1097/00001813-199506000-00001

[bib7] Blaney SM, Bernstein M, Neville K, Ginsberg J, Kitchen B, Horton T, Berg SL, Krailo M, Adamson PC (2004) Phase I study of the proteasome inhibitor bortezomib in pediatric patients with refractory solid tumors: a Children's Oncology Group study (ADVL0015). J Clin Oncol 22: 4804–4809 15570082 10.1200/JCO.2004.12.185

[bib8] Boccadoro M, Morgan G, Cavenagh J (2005) Preclinical evaluation of the proteasome inhibitor bortezomib in cancer therapy. Cancer Cell Int 5: 18 15929791 10.1186/1475-2867-5-18 PMC1164423

[bib9] Cardoso F, Durbecq V, Laes JF, Badran B, Lagneaux L, Bex F, Desmedt C, Willard-Gallo K, Ross JS, Burny A, Piccart M, Sotiriou C (2006) Bortezomib (PS-341, Velcade) increases the efficacy of trastuzumab (Herceptin) in HER-2-positive breast cancer cells in a synergistic manner. Mol Cancer Ther 5: 3042–3051 17148762 10.1158/1535-7163.MCT-06-0104

[bib10] Chan S, Romieu G, Huober J (2005) Gemcitabine plus docetaxel (GD) *vs* capecitabine plus docetaxel (CD) for anthracycline-pretreated metastatic breast cancer (MBC) patients (pts): results of a European phase III study. J Clin Oncol 23: 24s [Abstract] 10.1200/JCO.2007.15.8485 19273714

[bib11] Codony-Servat J, Tapia MA, Bosch M, Oliva C, Domingo-Domenech J, Mellado B, Rolfe M, Ross JS, Gascon P, Rovira A, Albanell J (2006) Differential cellular and molecular effects of bortezomib, a proteasome inhibitor, in human breast cancer cells. Mol Cancer Ther 5: 665–675 16546981 10.1158/1535-7163.MCT-05-0147

[bib12] Colson K, Doss DS, Swift R, Tariman J, Thomas TE (2004) Bortezomib, a newly approved proteasome inhibitor for the treatment of multiple myeloma: nursing implications. Clin J Oncol Nurs 8: 473–480 15515281 10.1188/04.CJON.473-480

[bib13] Cusack JC (2003) Rationale for the treatment of solid tumors with the proteasome inhibitor bortezomib. Cancer Treat Rev 29: 21–31 10.1016/s0305-7372(03)00079-3 12738240

[bib14] Dreicer R, Petrylak D, Agus D, Webb I, Roth B (2007) Phase I/II study of bortezomib plus docetaxel in patients with advanced androgen-independent prostate cancer. Clin Cancer Res 13: 1208–1215 17317831 10.1158/1078-0432.CCR-06-2046

[bib15] Dy GK, Thomas JP, Wilding G, Bruzek L, Mandrekar S, Erlichman C, Alberti D, Binger K, Pitot HC, Alberts SR, Hanson LJ, Marnocha R, Tutsch K, Kaufmann SH, Adjei AA (2005) A phase I and pharmacologic trial of two schedules of the proteasome inhibitor, PS-341 (bortezomib, velcade), in patients with advanced cancer. Clin Cancer Res 11: 3410–3416 15867242 10.1158/1078-0432.CCR-04-2068

[bib16] Eisenhauer EA, Vermorken JB (1998) The taxoids. Comparative clinical pharmacology and therapeutic potential. Drugs 55: 5–30 9463787 10.2165/00003495-199855010-00002

[bib17] Fanucchi MP, Fossella FV, Belt R, Natale R, Fidias P, Carbone DP, Govindan R, Raez LE, Robert F, Ribeiro M, Akerley W, Kelly K, Limentani SA, Crawford J, Reimers HJ, Axelrod R, Kashala O, Sheng S, Schiller JH (2006) Randomized phase II study of bortezomib alone and bortezomib in combination with docetaxel in previously treated advanced non-small-cell lung cancer. J Clin Oncol 24: 5025–5033 17075122 10.1200/JCO.2006.06.1853

[bib18] Farneth NC, Holland WS, Kenosi T, Kimura T, Lara PN, Gandara DR, Gumerlock PH (2005) Proteasome inhibition with bortezomib (PS-341) in combination with docetaxel (Doc) in prostate cancer (CaP) cells and xenografts. J Clin Oncol 23: 239s [Abstract]

[bib19] Gumerlock PH, Kawaguchi T, Moisan LP, Lau AH, Mack PC, Lara Jr PN, Gandara DR (2002) Mechanisms of enhanced cytotoxicity from docetaxel → PS-341 combination in non-small cell lung carcinoma (NSCLC). Proc Am Soc Clin Oncol 21: 304a [Abstract]

[bib20] Gumerlock PH, Kimura T, Holland WS, Shih CD, Lara Jr PN., Gandara DR (2004) Differential *in vivo* activity of docetaxel plus PS-341 combination therapy in non-small cell lung carcinoma (NSCLC) xenografts. J Clin Oncol 22: 649s [Abstract]

[bib21] Hainsworth JD, Yardley DA, Spigel DR, Meluch AA, Rinaldi D, Schnell FM, Greco FA (2006) Docetaxel and epirubicin as first-line treatment for patients with metastatic breast cancer: a Minnie Pearl Cancer Research Network Phase II trial. Cancer Invest 24: 469–473 16939953 10.1080/07357900600814060

[bib22] Hamilton AL, Eder JP, Pavlick AC, Clark JW, Liebes L, Garcia-Carbonero R, Chachoua A, Ryan DP, Soma V, Farrell K, Kinchla N, Boyden J, Yee H, Zeleniuch-Jacquotte A, Wright J, Elliott P, Adams J, Muggia FM (2005) Proteasome inhibition with bortezomib (PS-341): a phase I study with pharmacodynamic end points using a day 1 and day 4 schedule in a 14-day cycle. J Clin Oncol 23: 6107–6116 16135477 10.1200/JCO.2005.01.136

[bib23] Hideshima T, Richardson P, Chauhan D, Palombella VJ, Elliott PJ, Adams J, Anderson KC (2001) The proteasome inhibitor PS-341 inhibits growth, induces apoptosis, and overcomes drug resistance in human multiple myeloma cells. Cancer Res 61: 3071–3076 11306489

[bib24] Ikezoe T, Yang Y, Saito T, Koeffler HP, Taguchi H (2004) Proteasome inhibitor PS-341 down-regulates prostate-specific antigen (PSA) and induces growth arrest and apoptosis of androgen-dependent human prostate cancer LNCaP cells. Cancer Sci 95: 271–275 15016328 10.1111/j.1349-7006.2004.tb02215.x PMC11158246

[bib25] Jones SE, Erban J, Overmoyer B, Budd GT, Hutchins L, Lower E, Laufman L, Sundaram S, Urba WJ, Pritchard KI, Mennel R, Richards D, Olsen S, Meyers ML, Ravdin PM (2005) Randomized phase III study of docetaxel compared with paclitaxel in metastatic breast cancer. J Clin Oncol 23: 5542–5551 16110015 10.1200/JCO.2005.02.027

[bib26] Kandioler-Eckersberger D, Ludwig C, Rudas M, Kappel S, Janschek E, Wenzel C, Schlagbauer-Wadl H, Mittlbock M, Gnant M, Steger G, Jakesz R (2000) TP53 mutation and p53 overexpression for prediction of response to neoadjuvant treatment in breast cancer patients. Clin Cancer Res 6: 50–56 10656431

[bib27] Kane RC, Farrell AT, Sridhara R, Pazdur R (2006) United States Food and Drug Administration approval summary: bortezomib for the treatment of progressive multiple myeloma after one prior therapy. Clin Cancer Res 12: 2955–2960 16707588 10.1158/1078-0432.CCR-06-0170

[bib28] Lara Jr P, Koczywas M, Quinn D, Lenz H, Davies A, Lau D, Gumerlock P, Longmate J, Doroshow J, Schenkein D, Kashala O, Gandara D (2006) Bortezomib plus docetaxel in advanced non-small cell lung cancer and other solid tumors: a phase I California Cancer Consortium trial. J Thorac Oncol 1: 126–134 17409841

[bib29] Lenz HJ (2003) Clinical update: proteasome inhibitors in solid tumors. Cancer Treat Rev 29: 41–48 12738242 10.1016/s0305-7372(03)00082-3

[bib30] Lightcap ES, McCormack TA, Pien CS, Chau V, Adams J, Elliott PJ (2000) Proteasome inhibition measurements: clinical application. Clin Chem 46: 673–683 10794750

[bib31] Lin YC, Chang HK, Chen JS, Wang HM, Yang TS, Liaw CC (2007) A phase II randomized study of two taxanes and cisplatin for metastatic breast cancer after anthracycline: a final analysis. Jpn J Clin Oncol 37: 23–29 17172351 10.1093/jjco/hyl124

[bib32] Lonial S, Richardson P, Sonneveld P, Schuster M, Irwin D, Stadtmauer E, Facon T, Harousseau J, Ben-Yehuda D, Goldschmidt H, Reece D, San MJ, Blade J, Boccadoro M, Cavenagh J, Dalton W, Boral A, Schenkein D, Anderson KC (2005) Hematologic profiles in the phase 3 APEX trial. Blood 106: 970a [Abstract]

[bib33] Mack PC, Davies AM, Lara PN, Gumerlock PH, Gandara DR (2003) Integration of the proteasome inhibitor PS-341 (Velcade) into the therapeutic approach to lung cancer. Lung Cancer 41: S89–S96 12867067 10.1016/s0169-5002(03)00149-1

[bib34] MacLaren AP, Chapman RS, Wyllie AH, Watson CJ (2001) p53-dependent apoptosis induced by proteasome inhibition in mammary epithelial cells. Cell Death Differ 8: 210–218 11319603 10.1038/sj.cdd.4400801

[bib35] Maki RG, Kraft AS, Scheu K, Yamada J, Wadler S, Antonescu CR, Wright JJ, Schwartz GK (2005) A multicenter phase II study of bortezomib in recurrent or metastatic sarcomas. Cancer 103: 1431–1438 15739208 10.1002/cncr.20968

[bib36] Meluch A, Spigel D, Greco F, Barton J, Messina G, Gould B, Rovito M, Hainsworth J (2005) Weekly docetaxel and bortezomib in the treatment of patients with advanced hormone refractory prostate cancer (HRPC): a Minnie Pearl Cancer Research Network phase II trial. J Clin Oncol 23: 436 [Abstract] 10.3816/CGC.2007.n.004 17553208

[bib37] Messersmith WA, Baker SD, Lassiter L, Sullivan RA, Dinh K, Almuete VI, Wright JJ, Donehower RC, Carducci MA, Armstrong DK (2006) Phase I trial of bortezomib in combination with docetaxel in patients with advanced solid tumors. Clin Cancer Res 12: 1270–1275 16489083 10.1158/1078-0432.CCR-05-1942

[bib38] Millennium Pharmaceuticals Inc. Data on file

[bib39] Nabholtz JM, Senn HJ, Bezwoda WR, Melnychuk D, Deschenes L, Douma J, Vandenberg TA, Rapoport B, Rosso R, Trillet-Lenoir V, Drbal J, Molino A, Nortier JW, Richel DJ, Nagykalnai T, Siedlecki P, Wilking N, Genot JY, Hupperets PS, Pannuti F, Skarlos D, Tomiak EM, Murawsky M, Alakl M, Aapro M (1999) Prospective randomized trial of docetaxel *vs* mitomycin plus vinblastine in patients with metastatic breast cancer progressing despite previous anthracycline-containing chemotherapy. 304 Study Group. J Clin Oncol 17: 1413–1424 10334526 10.1200/JCO.1999.17.5.1413

[bib40] Nawrocki ST, Bruns CJ, Harbison MT, Bold RJ, Gotsch BS, Abbruzzese JL, Elliott P, Adams J, McConkey DJ (2002) Effects of the proteasome inhibitor PS-341 on apoptosis and angiogenesis in orthotopic human pancreatic tumor xenografts. Mol Cancer Ther 1: 1243–1253 12516957

[bib41] Nawrocki ST, Sweeney-Gotsch B, Takamori R, McConkey DJ (2004) The proteasome inhibitor bortezomib enhances the activity of docetaxel in orthotopic human pancreatic tumor xenografts. Mol Cancer Ther 3: 59–70 14749476

[bib42] O'Shaughnessy J, Miles D, Vukelja S, Moiseyenko V, Ayoub JP, Cervantes G, Fumoleau P, Jones S, Lui WY, Mauriac L, Twelves C, Van Hazel G, Verma S, Leonard R (2002) Superior survival with capecitabine plus docetaxel combination therapy in anthracycline-pretreated patients with advanced breast cancer: phase III trial results. J Clin Oncol 20: 2812–2823 12065558 10.1200/JCO.2002.09.002

[bib43] Orlowski RZ, Dees EC (2003) The role of the ubiquitination-proteasome pathway in breast cancer: applying drugs that affect the ubiquitin-proteasome pathway to the therapy of breast cancer. Breast Cancer Res 5: 1–7 12559038 10.1186/bcr460 PMC154126

[bib44] Orlowski RZ, Stinchcombe TE, Mitchell BS, Shea TC, Baldwin AS, Stahl S, Adams J, Esseltine DL, Elliott PJ, Pien CS, Guerciolini R, Anderson JK, pcik-Smith ND, Bhagat R, Lehman MJ, Novick SC, O'Connor OA, Soignet SL (2002) Phase I trial of the proteasome inhibitor PS-341 in patients with refractory hematologic malignancies. J Clin Oncol 20: 4420–4427 12431963 10.1200/JCO.2002.01.133

[bib45] Osin PP, Lakhani SR (1999) The pathology of familial breast cancer: immunohistochemistry and molecular analysis. Breast Cancer Res 1: 36–40 11250681 10.1186/bcr11 PMC138499

[bib46] Overgaard J, Yilmaz M, Guldberg P, Hansen LL, Alsner J (2000) TP53 mutation is an independent prognostic marker for poor outcome in both node-negative and node-positive breast cancer. Acta Oncol 39: 327–333 10987229 10.1080/028418600750013096

[bib47] Papandreou CN, Daliani DD, Nix D, Yang H, Madden T, Wang X, Pien CS, Millikan RE, Tu SM, Pagliaro L, Kim J, Adams J, Elliott P, Esseltine D, Petrusich A, Dieringer P, Perez C, Logothetis CJ (2004) Phase I trial of the proteasome inhibitor bortezomib in patients with advanced solid tumors with observations in androgen-independent prostate cancer. J Clin Oncol 22: 2108–2121 15169797 10.1200/JCO.2004.02.106

[bib48] Pharoah PD, Day NE, Caldas C (1999) Somatic mutations in the p53 gene and prognosis in breast cancer: a meta-analysis. Br J Cancer 80: 1968–1973 10471047 10.1038/sj.bjc.6690628 PMC2363143

[bib49] Rosing H, Lustig V, van Warmerdam LJ, Huizing MT, ten Bokkel Huinink WW, Schellens JH, Rodenhuis S, Bult A, Beijnen JH (2000) Pharmacokinetics and metabolism of docetaxel administered as a 1-h intravenous infusion. Cancer Chemother Pharmacol 45: 213–218 10663639 10.1007/s002800050032

[bib50] Shah MH, Young D, Kindler HL, Webb I, Kleiber B, Wright J, Grever M (2004) Phase II study of the proteasome inhibitor bortezomib (PS-341) in patients with metastatic neuroendocrine tumors. Clin Cancer Res 10: 6111–6118 15447997 10.1158/1078-0432.CCR-04-0422

[bib51] Shah SA, Potter MW, McDade TP, Ricciardi R, Perugini RA, Elliott PJ, Adams J, Callery MP (2001) 26S proteasome inhibition induces apoptosis and limits growth of human pancreatic cancer. J Cell Biochem 82: 110–122 11400168 10.1002/jcb.1150

[bib52] Sjostrom J, Blomqvist C, Mouridsen H, Pluzanska A, Ottosson-Lonn S, Bengtsson NO, Ostenstad B, Mjaaland I, Palm-Sjovall M, Wist E, Valvere V, Anderson H, Bergh J (1999) Docetaxel compared with sequential methotrexate and 5-fluorouracil in patients with advanced breast cancer after anthracycline failure: a randomised phase III study with crossover on progression by the Scandinavian Breast Group. Eur J Cancer 35: 1194–1201 10615229 10.1016/s0959-8049(99)00122-7

[bib53] Sunwoo JB, Chen Z, Dong G, Yeh N, Crowl Bancroft C, Sausville E, Adams J, Elliott P, Van Waes C (2001) Novel proteasome inhibitor PS-341 inhibits activation of nuclear factor-kappa B, cell survival, tumor growth, and angiogenesis in squamous cell carcinoma. Clin Cancer Res 7: 1419–1428 11350913

[bib54] Tapia M, Codony-Servat J, Domingo-Domenech J, Ferrer B, Fernandez PL, Ross JS, Rolfe M, Gascon P, Rovira A, Albanell J (2005) Activity of bortezomib, a proteasome inhibitor, in breast cancer cells: associations with negative estrogen receptor and IKK/NF-kB expression. J Clin Oncol 23: 233s [Abstract]

[bib55] Teicher BA, Ara G, Herbst R, Palombella VJ, Adams J (1999) The proteasome inhibitor PS-341 in cancer therapy. Clin Cancer Res 5: 2638–2645 10499643

[bib56] Therasse P, Arbuck SG, Eisenhauer EA, Wanders J, Kaplan RS, Rubinstein L, Verweij J, Van Glabbeke M, van Oosterom AT, Christian MC, Gwyther SG (2000) New guidelines to evaluate the response to treatment in solid tumors. European Organization for Research and Treatment of Cancer, National Cancer Institute of the United States, National Cancer Institute of Canada. J Natl Cancer Inst 92: 205–216 10655437 10.1093/jnci/92.3.205

[bib57] Vogel CL, Cobleigh MA, Tripathy D, Gutheil JC, Harris LN, Fehrenbacher L, Slamon DJ, Murphy M, Novotny WF, Burchmore M, Shak S, Stewart SJ, Press M (2002) Efficacy and safety of trastuzumab as a single agent in first-line treatment of HER2-overexpressing metastatic breast cancer. J Clin Oncol 20: 719–726 11821453 10.1200/JCO.2002.20.3.719

[bib58] Williams S, Pettaway C, Song R, Papandreou C, Logothetis C, McConkey DJ (2003) Differential effects of the proteasome inhibitor bortezomib on apoptosis and angiogenesis in human prostate tumor xenografts. Mol Cancer Ther 2: 835–843 14555702

